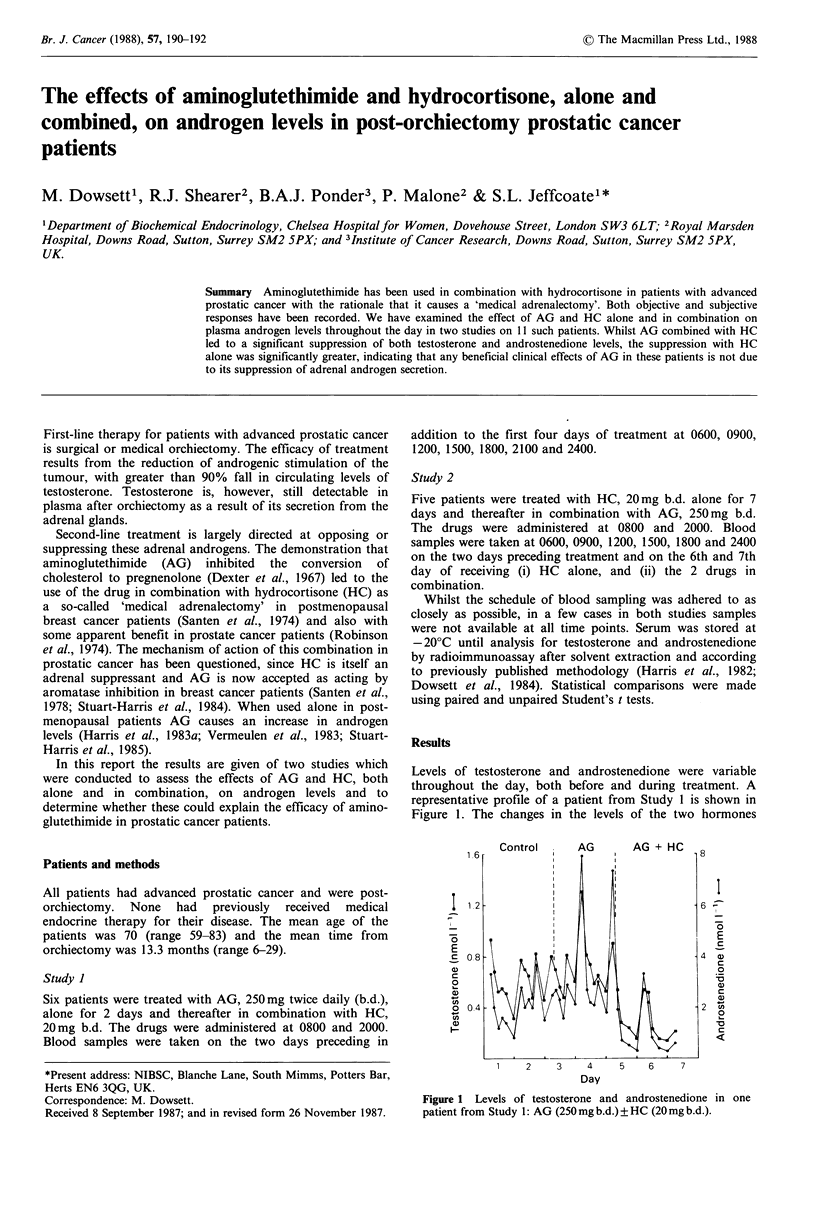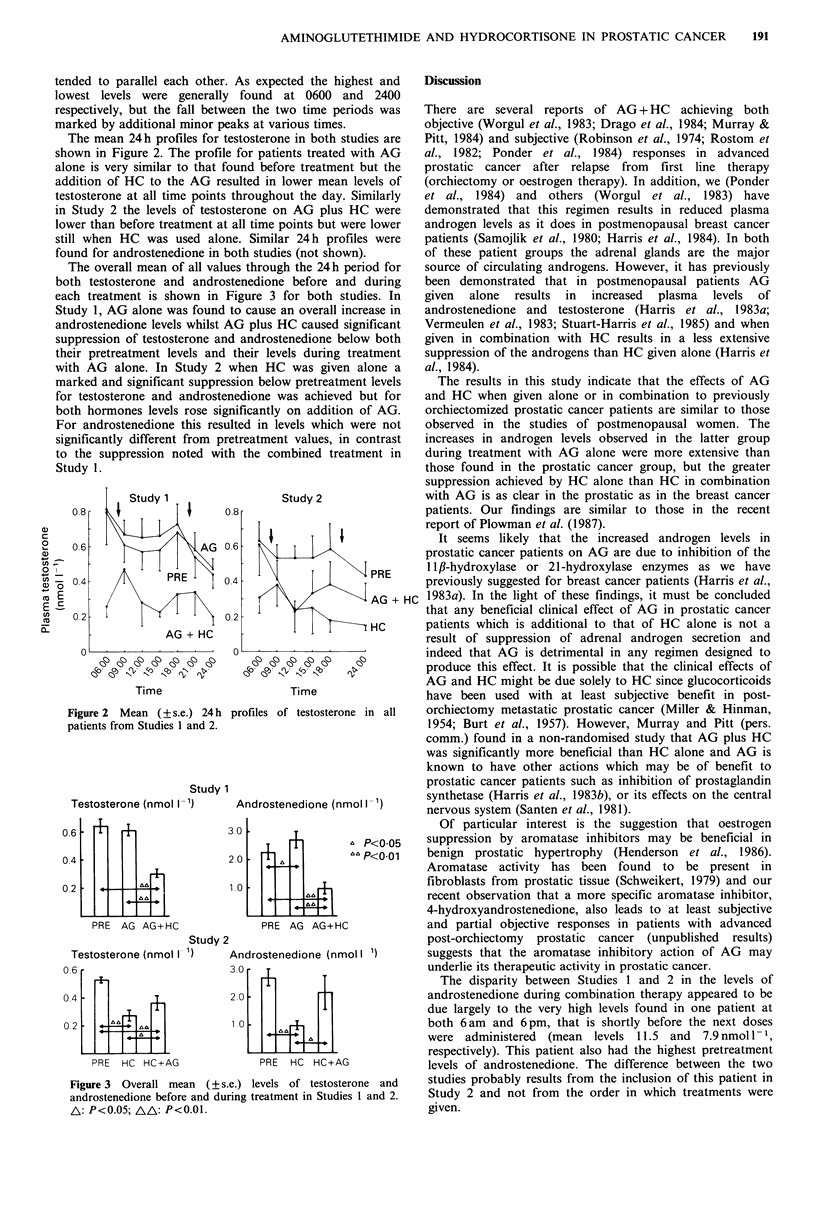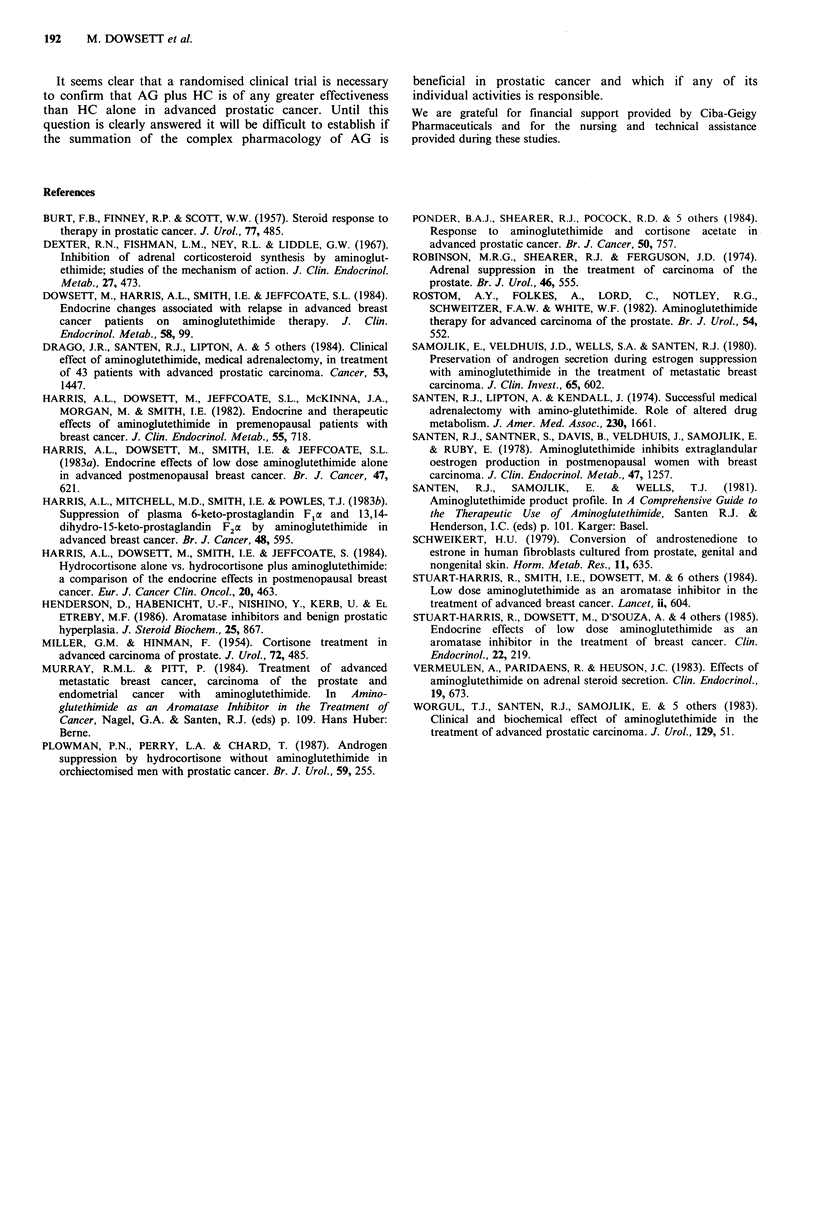# The effects of aminoglutethimide and hydrocortisone, alone and combined, on androgen levels in post-orchiectomy prostatic cancer patients.

**DOI:** 10.1038/bjc.1988.40

**Published:** 1988-02

**Authors:** M. Dowsett, R. J. Shearer, B. A. Ponder, P. Malone, S. L. Jeffcoate

**Affiliations:** Department of Biochemical Endocrinology, Chelsea Hospital for Women, London, UK.

## Abstract

Aminoglutethimide has been used in combination with hydrocortisone in patients with advanced prostatic cancer with the rationale that it causes a 'medical adrenalectomy'. Both objective and subjective responses have been recorded. We have examined the effect of AG and HC alone and in combination on plasma androgen levels throughout the day in two studies on 11 such patients. Whilst AG combined with HC led to a significant suppression of both testosterone and androstenedione levels, the suppression with HC alone was significantly greater, indicating that any beneficial clinical effects of AG in these patients is not due to its suppression of adrenal androgen secretion.


*Br. J. Cancer* (1988), **57**, 190–192 © The Macmillan Press Ltd., 1988

### The effects of aminoglutethimide and hydrocortisone, alone and combined, on androgen levels in post-orchiectomy prostatic cancer patients

M. Dowsett[1], R.J. Shearer[2], B.A.J. Ponder[3], P. Malone[2] & S.L. Jeffcoate[1]*

[1]*Department of Biochemical Endocrinology, Chelsea Hospital for Women, Dovehouse Street, London SW3 6LT;* [2]*Royal Marsden Hospital, Downs Road, Sutton, Surrey SM2 5PX; and* [3]*Institute of Cancer Research, Downs Road, Sutton, Surrey SM2 5PX, UK.*

**Summary** Aminoglutethimide has been used in combination with hydrocortisone in patients with advanced prostatic cancer with the rationale that it causes a 'medical adrenalectomy'. Both objective and subjective responses have been recorded. We have examined the effect of AG and HC alone and in combination on plasma androgen levels throughout the day in two studies on 11 such patients. Whilst AG combined with HC led to a significant suppression of both testosterone and androstenedione levels, the suppression with HC alone was significantly greater, indicating that any beneficial clinical effects of AG in these patients is not due to its suppression of adrenal androgen secretion.

First-line therapy for patients with advanced prostatic cancer is surgical or medical orchiectomy. The efficacy of treatment results from the reduction of androgenic stimulation of the tumour, with greater than 90% fall in circulating levels of testosterone. Testosterone is, however, still detectable in plasma after orchiectomy as a result of its secretion from the adrenal glands.

Second-line treatment is largely directed at opposing or suppressing these adrenal androgens. The demonstration that aminoglutethimide (AG) inhibited the conversion of cholesterol to pregnenolone (Dexter *et al.*, 1967) led to the use of the drug in combination with hydrocortisone (HC) as a so-called 'medical adrenalectomy' in postmenopausal breast cancer patients (Santen *et al.*, 1974) and also with some apparent benefit in prostate cancer patients (Robinson *et al.*, 1974). The mechanism of action of this combination in prostatic cancer has been questioned, since HC is itself an adrenal suppressant and AG is now accepted as acting by aromatase inhibition in breast cancer patients (Santen *et al.*, 1978; Stuart-Harris *et al.*, 1984). When used alone in post-menopausal patients AG causes an increase in androgen levels (Harris *et al.*, 1983*a*; Vermeulen *et al.*, 1983; Stuart-Harris *et al.*, 1985).

In this report the results are given of two studies which were conducted to assess the effects of AG and HC, both alone and in combination, on androgen levels and to determine whether these could explain the efficacy of aminoglutethimide in prostatic cancer patients.

### Patients and methods

All patients had advanced prostatic cancer and were post-orchiectomy. None had previously received medical endocrine therapy for their disease. The mean age of the patients was 70 (range 59–83) and the mean time from orchiectomy was 13.3 months (range 6–29).

### *Study 1*

Six patients were treated with AG, 250 mg twice daily (b.d.), alone for 2 days and thereafter in combination with HC, 20 mg b.d. The drugs were administered at 0800 and 2000. Blood samples were taken on the two days preceding in addition to the first four days of treatment at 0600, 0900, 1200, 1500, 1800, 2100 and 2400.

### *Study 2*

Five patients were treated with HC, 20 mg b.d. alone for 7 days and thereafter in combination with AG, 250 mg b.d. The drugs were administered at 0800 and 2000. Blood samples were taken at 0600, 0900, 1200, 1500, 1800 and 2400 on the two days preceding treatment and on the 6th and 7th day of receiving (i) HC alone, and (ii) the 2 drugs in combination.

Whilst the schedule of blood sampling was adhered to as closely as possible, in a few cases in both studies samples were not available at all time points. Serum was stored at −20°C until analysis for testosterone and androstenedione by radioimmunoassay after solvent extraction and according to previously published methodology (Harris *et al.*, 1982; Dowsett *et al.*, 1984). Statistical comparisons were made using paired and unpaired Student's *t* tests.

### Results

Levels of testosterone and androstenedione were variable throughout the day, both before and during treatment. A representative profile of a patient from Study 1 is shown in Figure 1. The changes in the levels of the two hormones

**Figure 1** Levels of testosterone and androstenedione in one patient from Study 1: AG (250 mg b.d.) ± HC (20 mg b.d.).

*Present address: NIBSC, Blanche Lane, South Mimms, Potters Bar, Herts EN6 3QG, UK.
Correspondence: M. Dowsett.
Received 8 September 1987; and in revised form 26 November 1987.

tended to parallel each other. As expected the highest and lowest levels were generally found at 0600 and 2400 respectively, but the fall between the two time periods was marked by additional minor peaks at various times.

The mean 24 h profiles for testosterone in both studies are shown in Figure 2. The profile for patients treated with AG alone is very similar to that found before treatment but the addition of HC to the AG resulted in lower mean levels of testosterone at all time points throughout the day. Similarly in Study 2 the levels of testosterone on AG plus HC were lower than before treatment at all time points but were lower still when HC was used alone. Similar 24 h profiles were found for androstenedione in both studies (not shown).

The overall mean of all values through the 24 h period for both testosterone and androstenedione before and during each treatment is shown in Figure 3 for both studies. In Study 1, AG alone was found to cause an overall increase in androstenedione levels whilst AG plus HC caused significant suppression of testosterone and androstenedione below both their pretreatment levels and their levels during treatment with AG alone. In Study 2 when HC was given alone a marked and significant suppression below pretreatment levels for testosterone and androstenedione was achieved but for both hormones levels rose significantly on addition of AG. For androstenedione this resulted in levels which were not significantly different from pretreatment values, in contrast to the suppression noted with the combined treatment in Study 1.

**Figure 2** Mean (±s.e.) 24 h profiles of testosterone in all patients from Studies 1 and 2.

**Figure 3** Overall mean (±s.e.) levels of testosterone and androstenedione before and during treatment in Studies 1 and 2. △: $P<0.05$; △△: $P<0.01$.

### Discussion

There are several reports of AG+HC achieving both objective (Worgul *et al.*, 1983; Drago *et al.*, 1984; Murray & Pitt, 1984) and subjective (Robinson *et al.*, 1974; Rostom *et al.*, 1982; Ponder *et al.*, 1984) responses in advanced prostatic cancer after relapse from first line therapy (orchiectomy or oestrogen therapy). In addition, we (Ponder *et al.*, 1984) and others (Worgul *et al.*, 1983) have demonstrated that this regimen results in reduced plasma androgen levels as it does in postmenopausal breast cancer patients (Samojlik *et al.*, 1980; Harris *et al.*, 1984). In both of these patient groups the adrenal glands are the major source of circulating androgens. However, it has previously been demonstrated that in postmenopausal patients AG given alone results in increased plasma levels of androstenedione and testosterone (Harris *et al.*, 1983*a*; Vermeulen *et al.*, 1983; Stuart-Harris *et al.*, 1985) and when given in combination with HC results in a less extensive suppression of the androgens than HC given alone (Harris *et al.*, 1984).

The results in this study indicate that the effects of AG and HC when given alone or in combination to previously orchiectomized prostatic cancer patients are similar to those observed in the studies of postmenopausal women. The increases in androgen levels observed in the latter group during treatment with AG alone were more extensive than those found in the prostatic cancer group, but the greater suppression achieved by HC alone than HC in combination with AG is as clear in the prostatic as in the breast cancer patients. Our findings are similar to those in the recent report of Plowman *et al.* (1987).

It seems likely that the increased androgen levels in prostatic cancer patients on AG are due to inhibition of the 11$\beta$-hydroxylase or 21-hydroxylase enzymes as we have previously suggested for breast cancer patients (Harris *et al.*, 1983*a*). In the light of these findings, it must be concluded that any beneficial clinical effect of AG in prostatic cancer patients which is additional to that of HC alone is not a result of suppression of adrenal androgen secretion and indeed that AG is detrimental in any regimen designed to produce this effect. It is possible that the clinical effects of AG and HC might be due solely to HC since glucocorticoids have been used with at least subjective benefit in post-orchiectomy metastatic prostatic cancer (Miller & Hinman, 1954; Burt *et al.*, 1957). However, Murray and Pitt (pers. comm.) found in a non-randomised study that AG plus HC was significantly more beneficial than HC alone and AG is known to have other actions which may be of benefit to prostatic cancer patients such as inhibition of prostaglandin synthetase (Harris *et al.*, 1983*b*), or its effects on the central nervous system (Santen *et al.*, 1981).

Of particular interest is the suggestion that oestrogen suppression by aromatase inhibitors may be beneficial in benign prostatic hypertrophy (Henderson *et al.*, 1986). Aromatase activity has been found to be present in fibroblasts from prostatic tissue (Schweikert, 1979) and our recent observation that a more specific aromatase inhibitor, 4-hydroxyandrostenedione, also leads to at least subjective and partial objective responses in patients with advanced post-orchiectomy prostatic cancer (unpublished results) suggests that the aromatase inhibitory action of AG may underlie its therapeutic activity in prostatic cancer.

The disparity between Studies 1 and 2 in the levels of androstenedione during combination therapy appeared to be due largely to the very high levels found in one patient at both 6 am and 6 pm, that is shortly before the next doses were administered (mean levels 11.5 and 7.9 nmol l$^{-1}$, respectively). This patient also had the highest pretreatment levels of androstenedione. The difference between the two studies probably results from the inclusion of this patient in Study 2 and not from the order in which treatments were given.

It seems clear that a randomised clinical trial is necessary to confirm that AG plus HC is of any greater effectiveness than HC alone in advanced prostatic cancer. Until this question is clearly answered it will be difficult to establish if the summation of the complex pharmacology of AG is beneficial in prostatic cancer and which if any of its individual activities is responsible.

We are grateful for financial support provided by Ciba-Geigy Pharmaceuticals and for the nursing and technical assistance provided during these studies.

### References

BURT, F.B., FINNEY, R.P. & SCOTT, W.W. (1957). Steroid response to therapy in prostatic cancer. *J. Urol.*, **77**, 485.

DEXTER, R.N., FISHMAN, L.M., NEY, R.L. & LIDDLE, G.W. (1967). Inhibition of adrenal corticosteroid synthesis by aminoglutethimide; studies of the mechanism of action. *J. Clin. Endocrinol. Metab.*, **27**, 473.

DOWSETT, M., HARRIS, A.L., SMITH, I.E. & JEFFCOATE, S.L. (1984). Endocrine changes associated with relapse in advanced breast cancer patients on aminoglutethimide therapy. *J. Clin. Endocrinol. Metab.*, **58**, 99.

DRAGO, J.R., SANTEN, R.J., LIPTON, A. & 5 others (1984). Clinical effect of aminoglutethimide, medical adrenalectomy, in treatment of 43 patients with advanced prostatic carcinoma. *Cancer*, **53**, 1447.

HARRIS, A.L., DOWSETT, M., JEFFCOATE, S.L., McKINNA, J.A., MORGAN, M. & SMITH, I.E. (1982). Endocrine and therapeutic effects of aminoglutethimide in premenopausal patients with breast cancer. *J. Clin. Endocrinol. Metab.*, **55**, 718.

HARRIS, A.L., DOWSETT, M., SMITH, I.E. & JEFFCOATE, S.L. (1983*a*). Endocrine effects of low dose aminoglutethimide alone in advanced postmenopausal breast cancer. *Br. J. Cancer*, **47**, 621.

HARRIS, A.L., MITCHELL, M.D., SMITH, I.E. & POWLES, T.J. (1983*b*). Suppression of plasma 6-keto-prostaglandin $F_1\alpha$ and 13,14-dihydro-15-keto-prostaglandin $F_2\alpha$ by aminoglutethimide in advanced breast cancer. *Br. J. Cancer*, **48**, 595.

HARRIS, A.L., DOWSETT, M., SMITH, I.E. & JEFFCOATE, S. (1984). Hydrocortisone alone vs. hydrocortisone plus aminoglutethimide: a comparison of the endocrine effects in postmenopausal breast cancer. *Eur. J. Cancer Clin. Oncol.*, **20**, 463.

HENDERSON, D., HABENICHT, U.-F., NISHINO, Y., KERB, U. & EL ETREBY, M.F. (1986). Aromatase inhibitors and benign prostatic hyperplasia. *J. Steroid Biochem.*, **25**, 867.

MILLER, G.M. & HINMAN, F. (1954). Cortisone treatment in advanced carcinoma of prostate. *J. Urol.*, **72**, 485.

MURRAY, R.M.L. & PITT, P. (1984). Treatment of advanced metastatic breast cancer, carcinoma of the prostate and endometrial cancer with aminoglutethimide. In *Aminoglutethimide as an Aromatase Inhibitor in the Treatment of Cancer*, Nagel, G.A. & Santen, R.J. (eds) p. 109. Hans Huber: Berne.

PLOWMAN, P.N., PERRY, L.A. & CHARD, T. (1987). Androgen suppression by hydrocortisone without aminoglutethimide in orchiectomised men with prostatic cancer. *Br. J. Urol.*, **59**, 255.

PONDER, B.A.J., SHEARER, R.J., POCOCK, R.D. & 5 others (1984). Response to aminoglutethimide and cortisone acetate in advanced prostatic cancer. *Br. J. Cancer*, **50**, 757.

ROBINSON, M.R.G., SHEARER, R.J. & FERGUSON, J.D. (1974). Adrenal suppression in the treatment of carcinoma of the prostate. *Br. J. Urol.*, **46**, 555.

ROSTOM, A.Y., FOLKES, A., LORD, C., NOTLEY, R.G., SCHWEITZER, F.A.W. & WHITE, W.F. (1982). Aminoglutethimide therapy for advanced carcinoma of the prostate. *Br. J. Urol.*, **54**, 552.

SAMOJLIK, E., VELDHUIS, J.D., WELLS, S.A. & SANTEN, R.J. (1980). Preservation of androgen secretion during estrogen suppression with aminoglutethimide in the treatment of metastatic breast carcinoma. *J. Clin. Invest.*, **65**, 602.

SANTEN, R.J., LIPTON, A. & KENDALL, J. (1974). Successful medical adrenalectomy with amino-glutethimide. Role of altered drug metabolism. *J. Amer. Med. Assoc.*, **230**, 1661.

SANTEN, R.J., SANTNER, S., DAVIS, B., VELDHUIS, J., SAMOJLIK, E. & RUBY, E. (1978). Aminoglutethimide inhibits extraglandular oestrogen production in postmenopausal women with breast carcinoma. *J. Clin. Endocrinol. Metab.*, **47**, 1257.

SANTEN, R.J., SAMOJLIK, E. & WELLS, T.J. (1981). Aminoglutethimide product profile. In *A Comprehensive Guide to the Therapeutic Use of Aminoglutethimide*, Santen R.J. & Henderson, I.C. (eds) p. 101. Karger: Basel.

SCHWEIKERT, H.U. (1979). Conversion of androstenedione to estrone in human fibroblasts cultured from prostate, genital and nongenital skin. *Horm. Metab. Res.*, **11**, 635.

STUART-HARRIS, R., SMITH, I.E., DOWSETT, M. & 6 others (1984). Low dose aminoglutethimide as an aromatase inhibitor in the treatment of advanced breast cancer. *Lancet*, **ii**, 604.

STUART-HARRIS, R., DOWSETT, M., D'SOUZA, A. & 4 others (1985). Endocrine effects of low dose aminoglutethimide as an aromatase inhibitor in the treatment of breast cancer. *Clin. Endocrinol.*, **22**, 219.

VERMEULEN, A., PARIDAENS, R. & HEUSON, J.C. (1983). Effects of aminoglutethimide on adrenal steroid secretion. *Clin. Endocrinol.*, **19**, 673.

WORGUL, T.J., SANTEN, R.J., SAMOJLIK, E. & 5 others (1983). Clinical and biochemical effect of aminoglutethimide in the treatment of advanced prostatic carcinoma. *J. Urol.*, **129**, 51.